# Examining Caregiver-Resident Communication and Apathy in Dementia in Nursing Homes During the COVID-19 Pandemic

**DOI:** 10.1093/geroni/igab046.1202

**Published:** 2021-12-17

**Authors:** Ying-Ling Jao, Diane Berish, Yo-Jen Liao, Marie Boltz

**Affiliations:** 1 Pennsylvania State University, University Park, Pennsylvania, United States; 2 Penn State University, University Park, Pennsylvania, United States

## Abstract

This presentation shares lessons learned from conducting a study examining the impact of staff caregivers’ communication approach on apathy in residents with dementia in nursing homes. Due to COVID-19 restrictions, this study had to be paused and required major revisions to continue, which resulted in significant delays and increased expenses. Additionally, this study required in-person data collection and video recordings to capture staff caregivers’ communication with residents with dementia during caregiving activities. However, due to the pandemic, nursing home residents’ daily routines have been significantly changed, making it challenging to capture the nature of caregiver-resident interactions. Furthermore, using masks created unforeseen barriers for capturing communication between staff caregivers and residents including difficulties in identifying residents’ facial expressions, which are a vital component of assessing apathy. The presentation describes approaches to communication with founders, collaborators, and clinical sites and discusses strategies to recruit participants and conduct data collection.

